# The Role of Sepsis and Antisepsis in Medicine and the Importance of Oral Sepsis as Its Chief Cause

**Published:** 1911-12-15

**Authors:** Wm. Hunter

**Affiliations:** London, Eng.


### THE ROLE OF SEPSIS AND ANTISEPSIS IN MEDICINE AND THE IMPORTANCE OF ORAL SEPSIS AS ITS CHIEF CAUSE.

BY WM. HUNTER, M.D., LONDON, ENG.

NOTE—This is a liberal abstract of an address made by Dr. Hunter at McGill University, Montreal, Canada, October 3, 1910, and published in the *London Lancet*, January 14, 1911. This address has attained considerable notoriety, because a writer in *Current Literature* of August 1911, made a garbled and unfair review of it, which has been strenuously condemned by almost every Dental Journal in the country. We reprint the abstract not only in fairness to Dr. Hunter, but because it contains much that is true and good, which should be more carefully considered by our profession. We shall eliminate much of the first part of the address which is not pertinent and give only that part which has a bearing on dental practice.

Time was when the greatest honor that could be paid a surgeon, was to term him a brilliant operator, and to cite the rapidity with which he could complete a specified operation. The modern surgeon covets the title of being a clean or aseptic surgeon. Because he knows that with a knowledge and the strict practice of antiseptic surgery he will be successful.

The time is now coming—if it is not here, that the modern physician will seek to be known as an Antiseptic Physician, and with a better knowledge and practice of medical antisepsis he too will be successful. A knowledge of the principles and practice of antiseptic medicine is as essential and necessary to the physician, as is a knowledge of the principles and practice of antiseptic surgery to the modern surgeon.

### THE PRINCIPLES OF ANTISEPTIC SURGERY.

The word principles as here used is in common use in surgery and medicine, we speak of the principles of surgery, medicine, pathology, therapeutics, etc. In the sense so used, the word means the essential or principal or fundamental or entire range of characteristic qualities. It means not only the inherent facts, but also the logical deductions to be made from these known facts and peculiar behaviors, and the uses we make of the sum total of this knowledge.

If any of us were asked by the layman to give off hand a clear statement of the principles of antiseptic surgery, we probably could not give all the facts, and the whole chain of reasoning, observation and experience on which the practice of Antiseptic Surgery is based. We would probably reply that organisms introduced into the wounds cause fever, inflammation, suppuration, etc., and therefore the necessity for rigid surgical cleanliness, and the exibition of the elaborate surgical ritual in surgical practice.

The motives for Antiseptic Surgery are clear and obvious and are demonstrated by the surgeon in actual practice every day. They are omnipresent and mandatory. The surgeon lives in a very atmosphere of aseptic worship, he worships at her shrines, and places offerings of clean hands and a pure antiseptic conscience and behavior on her altars. Figuratively, if not actually, he sees written in flaming characters over the door of the operating theatre, the words; "Abandon Sepsis All Who Enter Here." And if this should escape him, even the janitor at the door reminds him to change his shoes, as the place which he enters is antiseptic territory.

Is the modern surgical ritual overdone? Are the ceremonials and environments excessive? None of us today dare to disregard or overlook them. They are in large degree educational, and no shrine of modern scientific medicine and practice appeals more to the imagination than the modern surgical operating theatre. In no department of the practice of modern medicine are humane services so faithfully and scrupulously rendered as in the modern surgical ampitheatre.

The object of this entire ritual of surgical practice is designed to prevent the access of extraneous micro-organisms into the wounds which the surgeon must necessarily make in his work of relief. To avoid such a catastrophe the surgeon takes every possible precaution that knowledge and experience dictates to him. No precaution is too minute, no duty too trivial to prevent the attainment of his object, and the true surgeon is no respector of persons or high places.

In earlier days surgical cleanliness was not particularly valued or attained. We did not understand the method of infection and have made many practical misapplications of antiseptic principles and agencies. Now we know that the special organisms which the surgeon most has to fear are not those of the air, but the Staphylococci and Streptococci on the patients body, on the skin, fingers or instruments used. He therefore takes the precaution to guard against not only the organisms of the room and its air, his instruments his personal inclinations and habits, but he takes especial care of the field of operation. The full force of his precautions is to avoid the introduction of septic organisms into his wounds by his own fingers or his assistant's or the instruments he uses. If a good surgeon he will attribute suppuration or infectious results to carlessness in some of these respects, or to the air of the operation theatre.

You are asked to observe that the entire effort of the modern surgeon is to prevent access of infectious organisms into the body as a result of his operations, or at least of a conveyance of such organisms from any other source to the seat of his wounds, as, for example, from the skin, the intestines, or other infected organs into tissues or organs where their presence might prove serious or fatal. His responsibility extends over this procedure only. If infection occurs in or through his operation then he feels keenly his responsibility. If the infectious organisms are already in the body and come into the field of his operation, he is only responsible that they are not carried to other uninfected tissues through his carelessness or bungling methods. If the organisms already exist in disease formed in the tissues, the surgeon may relieve the infected tissues, but he may not be able to disinfect the whole body, that will be the physicians task. He may at times overlook a serious infection in some tissue which does not express itself formidably, as in the mouth for instance, but which may be a constant and fruitful source of general or special infection.

### SEPSIS IN MEDICINE.

I have dwelt on the extreme ritualistic regard and treatment which the surgeon shows towards sepsis in his work, that I might contrast it with the attitude of the medical practitioner toward Staphylococcal and Streptococcal Sepsis in Medicines or medical treatment. This attitude is a very curious one. If a doctor were asked what it was he would probably say that it differed in no way from that shown toward it in surgery. He would say that he was as keen to eradicate sepsis and its sequellae as the surgeon; as for example; in blood poisonings and diseases of the air and alimentary tracts, the lungs, liver, brain and innumerable glands and other organs commonly treated medically. In fact he could say that medical treatment had so far advanced that men were devoting their lives to particular phases of medical treatment, and the attitude of these specialists was precisely the same as the surgeon in regard to disinfectant treatment, although it should be said that the practical application of the remedies was slower and more difficult. The surgeon has maintained that his work is not wholly confined to disinfection, but the highest type of modern surgery is preventive, and it is only recently that the medical therapeutist has adopted the idea of preventive medicine. Medical doctors believe that most infectious diseases of the body which they are called upon to treat are necessarily complicated with Empyema, and that these grave septic affections are often the result of general body ill-health or of special organic weaknesses. Viscious infectious micro-organisms are always present in the alimentary canal and under favoring circumstances they gain access to other organs and tissues where they become disease producers, and and when they over-reach the medical remedies they become surgical cases. The medical doctor limits his work to the treatment of the various acute and chronic ailments which cause ill health with no special or evident local cause which would call for surgical interference, ranging from teething to typhoid fever etc. They are so numerous and omnipresent that they have blinded the medical doctor to the importance of preventing infection or sepsis, so vital a matter with the surgeon. Because so many of the doctors' cases are apparently not septic in origin, he naturally is not so keen in anticipating and preventing this important etiological factor. It can not be questioned that many internal diseases are aggravated if not propagated by unfavorable conditions or environments which cannot be surgically eliminated. Sepsis in surgery, its action, prevention, or removal was the greatest principle of pathology and practice with which the surgeon had to deal, while sepsis in medicine was a causal factor of altogether subsidiary importance; it was in fact only one of many others, when present it was in most cases only a complication of other diseases, not its primary cause, it might be a terminal complication of some other more important disease.

It is with this conception of the sphere of activity and importance of sepsis in medicine that the results of my work for many years compel me to take issue. And I am prepared to say that the above commonly accepted conclusions regarding the importance of sepsis do not represent even approximately the actual pathological and clinical facts of the case.

In my clinical experience septic infection is without exception the most prevalent infection operating in medicine and is a most importamt and prevalent cause and complication of many medical diseases. Its ill effects are widespread and extend to all systems of the body. The relations between these effects and the sepsis that causes them are constantly overlooked, because the existence of the sepsis itself is overlooked. The chief seat of such sepsis is the oral cavity; and the sepsis itself, when noted, is erroneously regarded as the result of various conditions of ill-health with which it is associated, and not as it really is, an important cause of complication.

The causal connection between the sepsis and its ill-effects can be demonstrated by removing the sepsis and noting the striking effect upon the character, intensity or every existence of the illness; such medical diseases for instance as; general malais, bad complexion, indigestion, intestinal disturbances, persistent anemias, pharyngeal and glandular troubles in children, chronic rheumatism, etc. These effects are not always alike, they attack one system or another, and the degree of infection will vary in individuals and on occasions, chronic tuberculosis may affect in one case the glands in the neck, in another it will show itself in the joints, or the bones, or the lungs etc., or it may attack many places or the entire body. In like manner sepsis may attack the tonsils, or the glands in the neck, or it may appear in the blood causing anemia, purpuras, obscure fevers, or in the nervous system, or the kidneys, making in its total effect a grave condition of ill-health.

In my judgment sepsis in medicine ranks as the most prevalent and potent infective disease in the body and it deserves the special attention of the whole medical profession, at least to the extent that it has heretofore had the professions entire neglect. The responsibility for its early detection, and properly accrediting its influence must rest with the doctor who first encounters the case. If he fails to adequately consider its bearing on the disease the patient will not receive the advice or treatment to which he in all fairness is entitled.

### ORAL SEPSIS.

In the foregoing sketch of the chief spheres of the doctor's work and interests I have omitted any reference to one portion of the body which should constantly come under his observation, indeed, more frequently than any other,—the oral cavity. Cases such as I shall presently describe, could be multiplied by thousands and tens of thousands, and coming under the daily notice of doctors, who fail to take them into account, shows how constant this omission is in medical practice.

There is no part of the human body more commonly looked into by the doctor than the mouth. He looks at the tongue and sometimes at the gum tissue and gets symptomatic information as to disease in the internal organs. The tongue generally indicates a mal-condition of the intestinal tract or the liver, kidney, etc. and it gives the doctors reliable information as to obscure and inacessible organs.

When looking into the mouths of his patients the doctor has many occasions to note the prevalence of decayed or defective teeth, apparently an acccmpaniment of body ill-health, poor nutrition or same wasting disease. The defects are so common that most physicians cease to worry about them, especially since they have so often asked these patients how they can expect their digestion to be good with such teeth. What else can they expect but indigestion if they will not have their teeth put in good condition by the dentist, by treatment, or the supplying of lost ones. The doctor finds they will not take his advice on this matter and in some cases they actually resent such interference on the doctors' part, as they consider that a matter only between themselves and the dentist which can have no bearing on their general health, and consequently the doctor has come to depend on the dentist to look after this matter and does not associate poor teeth with body ailments.

Now I want to impress on all medical students, and practitioners as well, that it is not a matter of teeth and dentistry alone. It is an all important matter of sepsis and antisepsis that concerns every branch of the medical profession, and it concerns very closely the public health of the community. It is not a simple matter of neglect of the teeth by the patient, as is so commonly stated, but one of neglect of a far reaching infection by our profession; it is an infective disease for which the patient is not to be held entirely responsible, any more than he should be held responsible for contracting typhoid fever or tuberculosis. This is the condition I have called Oral Sepsis.

This title of Oral Sepsis I first called attention to in a paper entitled, "Oral Sepsis as a cause of Disease," which was published in the *British Medical Journal*, July, 1900. My object in giving this name, after mature deliberation and experience, was to emphasize the great fact, that it is not the absence of teeth, but the presence of sepsis; that it is not dental defects, but septic effects; that it is not defective mastication, but effective sepsis associated with such dental defects, that are responsible very often for much ill health associated with diseased mouths.

My second object was to emphasize the importance of the infections caused by Staphylococcal and Streptococcal organisms, as distinguished from the purely saprophytic infections in which the mouth abounds; or the occasional presence of specific organisms, such as typhoid, tubercle, pneumonia, etc.

Oral sepsis as I defined it, includes all septic lesions of Streptococcal and Staphylococcal infection found in the mouth, and it belongs to no one department of medicine more than another. It is common ground on which the general doctor, surgeon, the eye and ear, or nose and throat specialists, and last but not least the dental surgeons, all meet on terms of mutual and equal responsibility. A thorough grasp of the principles of antisepsis will be sufficient to control its effects in the earlier stages and manifestations, but unfortunately it is this knowledge that is lacking at the critical period.

One would think poorly of a surgeon or doctor who refused to treat a follicular tonsillitis, and insisted on referring all such cases to the throat specialists; or who permitted his patient to continuously suck a running sore on his finger. I think no less poorly of any doctor or surgeon who declines to make himself responsible for the treatment of much of the oral sepsis in the mouths of his patients. Wherein is the essential pathologic differences between a septic tonsillitis and a septic and suppurating condition of the gums, complicated with extensive calcular deposits, covering septic wounds and ulcers loaded with Staphylococci and Streptococci. The chief difference clinically is that gum suppuration is common while tonsillitis is comparatively rare. The pathologic results in both are sepsis. The sad fact is that while both are a serious health menace they are both amenable to the simplest treatment, and do not get it.

It is said that gum sepsis is so common and its evil effects so rare that it is futile to attempt its treatment. I have shown in my original paper, and my further research confirms my findings that the evil effects are both common and grave, and that no more serious consequences result only because of the powerful restraining influence of the buccal secretions. Oral sepsis is of urgent importance in relation to the whole widespread group of medical, surgical and dental affections caused by the actual presence or toxic action of these pyogenic organisms.

My clinical experience satisfies me that if oral and nasopharyngeal sepsis could be successfully excluded, the other channels, by which "medical sepsis" gains entrance into the body, might almost be ignored, and sepsis as an important and prevalent cause of disease in medicine would almost cease to exist, instead of being, in my opinion at the present time a more important and prevalent cause of disease in the domain of medicine than it is in surgery. The time was when sepsis was so prevalent in the surgical wards that is overflowed into the medical wards in the form of Amyloid diseases of the kidneys, liver and intestines, at the present time the most severe manifestation of sepsis and its effects are found in the medical wards, whence they overflow into the surgical wards as appendicitis, gall-bladder, pleuritic, and pyclitic inflammations and suppurations. It is of course impossible to keep all septic infection out of the mouth, just as it is impossible to prevent access of diphtheria, typhoid, pneumococcus and turbercle infections; but this should not deter us from taking rational precautions for these infections means out of the milk and water we drink or the air we breathe, similarly I am confident much of oral sepsis could be prevented.

I submit them, in the interests of the many sufferers from this great group of medical affect ons which produces that oral sepsis,. the chief channel of pyogenic affections, is deserving of ncreased notice and attention. Knowing as we do the pathogenic qualities of Staphylococci and Streptococci we should not have the slightest excuse for allowing the mouth, so easily accessible to local treatments, to remain the chief seat of its open wounds and a veritable hotbed for their development and propagation, and it will be a serious reflection on our profession to allow it to continue.

Even if we grant that dental caries is not a marked septic or infective disease except in rare cases where the pulpal tissue is involved,. the gums and periosteum and even the alveolus are often involved in septic degeneration and so become sources of alimentary and venous infection.

In many cases there is little or no caries, but extensive suppuration of the gums and peridental structures, with deep pus pockets, discharging continually into the mouth. These conditions are easily observed by the physician and he should know that they are productive of some of the worst septic bodily diseases. It is true some of the worst cases are not so obvious until the teeth loosen and are exfoliated.

It is the poor people who are the great sufferers in most cases, as they give their mouths and teeth very little attention and their teeth become carious and the gums infected, until they are compelled to have them extracted because they are to uncomfortable.

But the problem presented by similar conditions in private patients is a much more difficult one. One of the worst cases of sepsis I have ever seen was brought to me by a doctor, who told me the mouth had been carefully seen to and was in good order, the patient was a tall handsome man in the prime of life, with a severe case of Addison's Anemia. His mouth was indeed clean to all appearances, but it was one mass ot gold caps, bridges, crowns, fillings and porcelain teeth, so ingeniously built up that one could scarcely tell what was false and what was real. To free this patient from sepsis in his state of health involved what was really equivalent to a major operation in surgery. The conditions of sepsis, and necrosis revealed on removing this golden architecture were perfectly appalling.

No one has probably had more reason than I have had to admire the shear ingenuity and mechanical skill constantly displayed by the dental surgeon. And no one has had more reason to appreciate the ghastly tragedies of oral sepsis which his misplaced ingenuity so often carries in its train. Gold fillings, crowns and bridges, fixed dentures, built on and about diseased tooth roots form a veritable mausoleum over a mass of sepsis to which there is no parallel in the whole realm of medicine and surgery. A perfect gold trap of sepsis of which the patient is the proud owner and no persuasion will induce him to part with it, for it has cost him much money and it covers his black and decayed teeth. There is no rank of society free from the fatal effects of health of this surgical malpractice.

The worst cases of anemia, gastritis, colitis, obscure fevers, nervous disturbances of all kinds from mental depression to actual lesions of the cord, chronic rheumatic affections, kidney diseases, are those which owe their origin to, or are gravely complicated by the oral sepsis produced by these gold traps of sepsis. Time and again I have traced the very first onset of the whole trouble to a period within a month or two of their insertion. This form of sepsis is particularly severe and injurious, because it is dammed up in the periosteum and alveolus and can not be eliminated by any ordinary medical antisepsis the doctor can administer, moreover it being locally painless and insidious in action, it goes on accumulating in severity without giving any symptom or warning.

Such are the fruits of this baneful so called conservative dentistry. The title would be fitting, if the teeth were a series of ivory pegs planted in stone sockets, but being highly developed and vital tissues, rich in blood and nerve supply, planted in a socket of bone with intimate vascular supply to the periosteum and gum tissue, a better title to such operations would be not conservative, but septic dentistry, Conservative it is, but only in one sense. It conserves the sepsis which it produces under the gold caps, by the satisfied patient, and the pride which the dentist feels in his "high class American work"and his unwillingness to recognize the sepsis which it produces.

There can be no doubt that the future of oral pathology and treatment, and of practical dentistry depends upon the extent to which those who occupy themselves with these subjects are trained in the principles, and become impregnated with the fundamental truths of oral sepsis and antisepsis. It is an important problem for solution by the dental profession in the interest of public health, especially of school children, 30 to 50 per cent. of whom suffer from dental and oral sepsis and its tonsillitic, pharyngeal, glandular and other affects. While a large portion of the dental profession is dealing successfully with the difficult problems of dental disease and oral sepsis, another portion is no less steadily engaged in promoting sepsis of the worst character and degree by ignoring the fundamental truths connected with the anatomy, physiology and pathology of the tissues with which they deal. To gold cap a diseased tooth in order to beautify it is the negation of every one of these sciences, and a veritable apotheosis of septic surgery, and of surgical and medical mal-practice.

The medical ill effects of this septic surgery are to be seen every day in those who are the victims of this gilded dentistry; their gray or sallow wax-like complexions, chronic dispepsias, intestinal disorders, anemias and neurotic complaints show it.

In no class of patients and in no country are these, in my observation, more common than among Americans and in America, the original home of this class of work.

### CLINICAL EFFECTS OF SEPSIS.

The chief feature of this particular oral sepsis is that the whole of it is swallowed or absorbed into the lymphatics and blood. Unlike sepsis of open wounds on the outside of the body none of it lost by external discharge. The full effect therefore falls first upon the alimentary tract from the tonsils and neck glands down through the whole tract and all organs associated with it, producing a long list of diseases, tonsillitis, pharyngitis, gastritis, dispepsia, enteritis and calitis, appendicitis etc., secondly it causes adenitis, septic anemia, purpura, fevers, septicemia, arthritis, nephritis and neurosis. The first principle involved is that this form of sepsis reaches the body by being swallowed or absorbed, and caries definite and deterious effects where found, with result varying according to vital resistance of the tissues invaded.

In the mouth on the healthy mucous surface this infectious material produces no injury any more than it would on the uninjured skin; but when the gum tissue is broken or injured infection rapidly takes place, and ultimately attacks all the peridental tissues. The mouth membranes are especially immune to ordinary infection which would readily attack internal tissues. The most intense anemia, blood poisoning, hectic fever, and ulcerative endocarditis may in my experience be produced by a septic tooth filled with amalgam, a deep seated alveolar abscess, or a suppurating autrum. On the other hand a chronic ulcer or abscess may not cause the slightest pyemia or inconvenience for a long time but sooner or later a bad sepsis will follow. It may not of itself produce a bad sepsis, but by co-operating with favoring conditions elsewhere deplorable results may ensue. A patient's illness does not always result from his chief disease, which may be incurable. The chief interest of clinical medicine, as distinguished from systemic medicine lies in excluding all the causal factors operating in any case and in removing as far as possible every adventitious factor, even if the chief one is beyond control,—as for instance a permanent organic disease.

### ILLUSTRATIVE CASES.

A man aged 32 was suffering from chronic indigestion and gastric trouble. His habits were good and his physique fine and apparently there was no reason why he should be so troubled. I found a tooth plate which had not been removed for two years, which he was told by his dentist he should not do. He finally removed it on my suggestion with considerable difficulty. There was much inflammation and ulceration under the plate and about several necrosed roots. I disinfected and applied an antiseptic dressing. After several days of similar treatment there was great local improvement, and after removing the abscessed roots the recovery was rapid and the patient was practically well in about two weeks. I call this "septic gastritis" from bad dentistry. Nine tenths of the cases of dispepsia and gastric trouble are either caused or complicated by similar oral sepsis, as they readily recover when these conditions are removed. Doctors are not looking for oral sepsis as a complication of systemic diseases.

A woman, age 33, presented a severe case of gastritis. She presented a broken down appearance and dirty gray complexion, a complete physicial examination, including a chemical and microscopic examination of the faeces. Her mouth showed grave oral sepsis from ulceration of the gums and extensive deposits of tartar on the teeth, covering pus pockets, and many of the teeth were exceedingly loose. She had been swallowing this infection for years, and it had caused septic gastritis and septic colitis, there was pus in the faeces. The doctor had observed and carefully recorded nearly every symptom, but had entirely overlooked the teeth.

Another patient came to the hospital suffering from chronic colitis, who had been treated without result in several other hospitals. He passed large masses of mucous with each motion. This patient had 20 tooth roots, the crowns having decayed away completely. These roots were badly abscessed and the patient had been swallowing quanties of mucous for many years. This I call "Septic Colitis." The roots were extracted the gums treated, and in four days no mucous was found in the stools and the patient's bowels became regular. In three weeks of this kind of treatment the patient was dismissed well, from a bad "Septic Colitis," caused by oral sepsis. If this case of Colitis had been caused originally by typhoid fever or dysentery it would have been seriously exaggerated by the oral sepsis.

A woman who had suffered for seven years from anemia, which was so marked that a celebrated physician warned her that it might become septic anemia if neglected. When she came to me she was an invalid from chronic anemia, weakness, palpitation, etc. She had tried all sorts of cures without success. For twelve years she had suffered from acute neuralgic pains on the right frontal region, followed by a discharge of pus from the nose. A chronic and foul empyema of the right frontal sinus, with an antral complication was discovered. It could not be said that these local lesions would cause the anemia, but they certainly had little restraining influence. The blood changes of haemoglobin satisfied me that it was "Septic Anemia" from the frontal sinus. The eradication of the diseased sinus was made and the patient left the hospital in a few weeks cured.

Dr. Hunter cites six or seven other cases of "Septic Anemia and Nephritis, with treatment and results similar to those quoted.

### ORAL SEPSIS AS A COMPLICATION OF SEPTIC FEVERS.

In 648 cases of scarlet fever admitted into the London Fever Hospital during the four years 1904 to1907, the incidence of oral sepsis, carefully noted by myself, varied from 25 to 43 per cent. The removal as far as was practicable immediately on reception, of every trace of oral sepsis by daily swabbing with a one to forty phenol solution, was very striking. The incidence of secondary adenitis was reduced from 9.6 per cent. in 1904 to 3.3 per cent., in 1906 and to 1.8 in 1907; of cellulitis of the neck, from 5.2 per cent. in 1904, to 2.8 per cent. in 1906, and to nothing in 1907; of glandular suppuration, from 1.7 per cent. in 1904 to 0.5 per Cent. in 1906, and to nothing in 1907. This marked improvement was caused by the increasing care taken to make the treatment. In only two cases were any teeth extracted.

### PRINCIPLES OF TREATMENT.

The first and most important matter is to make a most careful examination of mouth and naso-pharynx to detect any and all septic foci. This diagnosis can not be made hurridly or without knowledge of conditions to be observed. It will not be sufficient to note that the "teeth are fairly good," or that "the mouth needs attention." If you know where to look, you will see in many cases, every degree of septic gingivitis and ulceration; tartar deposits, loose and suppurating teeth and gums, septic ostitis, absorption of the gums, alveolar necrosis, and all forms of stomatitis. All this you can observe in much less time than is required to make any other physical diagnosis in the body. In particular cases, you will observe that these septic conditions are produced, or intensely aggravated by tooth plates, crowns, bridges, fillings, etc., which however good to begin with, are never really anti-septic, and are liable to become extremely septic.

The important matter is that you make a right diagnosis, and determine which are causes and which effects. The general course is first marginal infection, then peridental, and finally alveolar infection, resulting in inflammation, suppuration, and necrosis, with more or less general septic influence locally and systemically. The prime causal factor in all these processes is the septic infection, all the other processes are sequellae or results.

I grade the degree of sepsis found by degrees, from 1 to 10, one being very slight, while ten would indicate a maximum. If the teeth are all good except one or two which are slightly infected it would be graded mild or 1 or 2 degrees. Or if a patient presents with a mouth reeking with sepsis from a mass of bad crown and bridge work, or other misplaced and ill fitting dentures he would be entitled to a grade of 10 degrees. I have had many such cases, they are the most trying and pitable one has to deal with, they are really produced by the dentist. Some of my worst cases of septic gastritis, enteritis, colitis, ill health, anemia, obscure septic fevers, and other manifestations of medical sepsis; are in my experience those in which the greatest so called care in the way of American dental skill, in the constructing gold crowns and bridges had been used by the dentist. Patients always decline to have such work disturbed because, they have paid much money for it and they believe it could not be better done by anyone else.

In making my diagnosis I touch the gums with the phenol solution, which enables me to see the underlying structures, and it kills all superficial sepsis. The antiseptic treatment should be applied every day until complete resolution has taken place. It will be surprising how quickly patients will show marked improvement in health. This simple treatment is a great relief to the patient and prepares the mouth for further treatment by the doctor or dental surgeon. The patient learns from this treatment that much may be done for his oral health without painful surgical operations, and he also learns the value of careful oral treatment for the health of his mouth as well as his general system. In one case a patient consented to have ten or twelve roots extracted, who came very ill from a long siege of fever of obscure origin; and who recovered so rapidly as to surprise herself and the doctor who sent her to the hospital.

The fever which ranged from 101 to 103 degrees fell to normal the next day after the roots had been extracted.

The surroundings which I want to see provided in all general and dental hospitals and school clinics for providing this simple rescue and preventive treatment are less furniture and terrifying appliances that frighten and deter patients from applying for the treatment. The treatment must be carefully and intelligently applied by a competent person, who will reassure patients, and produce results that will convince them of its value and importance.

These principles are simple and effective, and are such as are used in surgical treatment. If they can be put into practice in the domain of medicine to prevent some of the more prevalent diseases of medical practice, as in my experience they can be, it becomes the duty of the profession to carry them into practice and see that ample provision is made in every hospital for caring for oral sepsis.

It may be said that the disease is so common that it is impossible to cope with it, but the same has been said of other pernicious and prevalent diseases which have been brought under control by medical science. To deal effectively with all uncleanly diseases and to teach people to be clean in all sanitary arrangements appeared to be a hopeless task when first proposed; but everything can be done in time, if the underlying principles are sound. So with the great evil of oral sepsis, and its manifold ill effects, far more widespread in their extent and no less severe in their character than those connected with typhoid fever or tuberculosis; the plea of helplessness to deal with it is a singularly poor one to be advanced or supported by the medical or surgical profession, at whose instigation the most elaborate and expensive equipments of hospitals and clinics are being provided.

